# COVID-19 Vaccine Effectiveness in Individuals with Alcohol and Tobacco Use Disorders: A Propensity Score-Matched Study Using Nationwide Brazilian Data

**DOI:** 10.3390/vaccines14050376

**Published:** 2026-04-23

**Authors:** Fabrício Emanuel S. Oliveira, Daniella R. B. Martelli, Maria Christina L. Oliveira, Enrico A. Colosimo, Ana Cristina Simões e Silva, Ana Livia O. Andrade, Rafaela R. Herrerias, Lays R. C. Foligno, Isabella O. Barbosa, Hercílio Martelli-Junior, Eduardo A. Oliveira

**Affiliations:** 1 Health Science/Primary Care Postgraduate Program, State University of Montes Claros (Unimontes), Montes Claros 39401-089, MG, Brazil; fabricioemanuel1@hotmail.com (F.E.S.O.); daniellareismartelli@yahoo.com.br (D.R.B.M.); hmjunior2000@yahoo.com (H.M.-J.); 2 Department of Pediatrics, Health Sciences Postgraduate Program, School of Medicine, University Federal of Minas Gerais (UFMG), R. Engenheiro Amaro Lanari 389/501, Belo Horizonte 30310-580, MG, Brazil; chrismariana@gmail.com (M.C.L.O.); acssilva@hotmail.com (A.C.S.e.S.); analiviaoliveira@ufmg.br (A.L.O.A.); rafaelarherrerias11@gmail.com (R.R.H.); laysribeiroc@ufmg.br (L.R.C.F.); isabellaob@ufmg.br (I.O.B.); 3 Department of Statistics, Federal University of Minas Gerais (UFMG), Belo Horizonte 31270-901, MG, Brazil; enricoc57@gmail.com

**Keywords:** alcoholism, smoking, COVID-19, COVID-19 vaccines, mortality, tobacco use disorder, alcohol use disorder

## Abstract

**Background/Objectives**: Individuals with alcohol use disorder (AUD) and tobacco use disorder (TUD) are at increased risk for severe COVID-19 outcomes. However, real-world evidence on vaccine effectiveness (VE) in these populations remains limited, particularly in low- and middle-income countries. This study aimed to evaluate the effectiveness of three or more COVID-19 vaccine doses against mortality in hospitalized patients with AUD and TUD in Brazil. **Methods**: This retrospective cohort study used data from the SIVEP Gripe database, a national surveillance system of hospitalized COVID-19 cases in Brazil. The study included adults aged ≥18 years with confirmed SARS-CoV 2 infection between February 2020 and June 2025. The intervention was defined as receipt of three or more vaccine doses (fully vaccinated) versus no doses (unvaccinated). Propensity score matching was performed separately for AUD and TUD cohorts. Vaccine effectiveness was estimated using McNemar’s test for paired samples, and the average treatment effect (ATE) and number needed to vaccinate (NNV) were calculated. **Results**: Among 2,184,723 hospitalized patients, 12,115 had AUD and 45,679 had TUD. After matching, VE against mortality was 42% (95% CI: 27.5–53.5) in the AUD group and 52.6% (95% CI: 46.5–58.1) in the TUD group, compared to 58.5% and 58.9% in their respective non-exposed counterparts. The ATE was consistent across groups (approximately −0.12), and the NNV to prevent one death was 8 (95% CI: 6–15 for AUD; 7–12 for TUD). **Conclusions**: Although VE was attenuated in individuals with AUD and TUD compared to the general population, the absolute benefit of vaccination remained substantial.

## 1. Introduction

The COVID-19 pandemic, caused by the SARS-CoV-2 virus, has posed an unprecedented global challenge to healthcare systems and revealed significant vulnerabilities in specific population subgroups. Among individuals at risk for severe outcomes, those with substance use disorders stand out, particularly Alcohol Use Disorder (AUD) and Tobacco Use Disorder (TUD). Population-based studies and clinical analyses have consistently demonstrated that these patients experience a more severe clinical course when infected with SARS-CoV-2 [1,2,3,4]. Both AUD and TUD are classified as mental health conditions characterized by problematic patterns of substance use that lead to clinically significant impairment or distress. The current biomedical framing emphasizes that these disorders involve dysfunction in the brain’s reward, motivation, and memory circuits, which leads to a characteristic loss of control and compulsive substance use despite negative consequences [5,6,7].

AUD and TUD are not merely behavioral risk factors but conditions that induce profound biological changes, compromising the immune response and integrity of physiological barriers. In AUD, chronic alcohol consumption impairs both innate and adaptive immunity by reducing alveolar macrophage function, disrupting the respiratory epithelial barrier, and decreasing the production of pro-inflammatory cytokines essential for viral clearance. Additionally, alcohol-induced gut dysbiosis and increased intestinal permeability leading to systemic endotoxemia, further exacerbating immune dysfunction. In TUD, chronic nicotine exposure compromises mucociliary clearance, damages the airway epithelium, and induces oxidative stress, whereas cigarette smoke components suppress natural killer cell activity and alter the antibody responses. These biological alterations result in increased susceptibility to respiratory infections and worse clinical outcomes. Consequently, patients with AUD have substantially higher risks of COVID-19 hospitalization, intensive care unit admission, and mortality [8,9].

Brazil’s vaccination program, launched in January 2021, faced extraordinary logistical and political challenges [10]. Following emergency use authorizations from the National Health Surveillance Agency (ANVISA), three vaccine platforms were approved: mRNA (Pfizer-BioNTech, New York, NY, USA), inactivated virus (Sinovac/CoronaVac, Beijing, China), and viral vector (ChAdOx1 nCoV-19, AstraZeneca, Cambridge, UK; Ad26.COV2.S, Janssen, Beerse, Belgium). The Ministry of Health (MS) served as the sole vaccine provider, requiring coordination across all levels of the decentralized Unified Health System (SUS) to reach a population of over 210 million. By mid-2021, fewer than 25% of the Brazilian population was fully vaccinated. During the first year of rollout, social vulnerability strongly predicted low COVID-19 vaccine coverage [11,12]. However, by the second half of 2021, distribution became markedly more equitable across regions and social strata, a temporal pattern that underscores the institutional capacity of the SUS and the National Immunization Program to reduce initial inequities, even amid federal disarray and supply constraints [13,14,15]. Nonetheless, vaccine hesitancy, fueled by misinformation and political polarization, persisted, leading to suboptimal coverage in certain demographic and regional subgroups [16]. This delayed and uneven rollout had important implications for vulnerable populations, including individuals with AUD and TUD, who faced both biological susceptibility and structural barriers to timely immunization [17,18,19].

Vaccination has been the primary strategy for mitigating the COVID-19 pandemic; however, its effectiveness may be modulated by chronic alcohol and tobacco use. While some early studies paradoxically suggested a protective effect of smoking against severe COVID-19 outcomes [20,21], emerging evidence indicates that tobacco use ultimately impairs humoral immune responses to vaccination [22]. Similarly, chronic alcohol consumption has been associated with reduced antibody levels and a faster decline in immunity following primary vaccination, although booster doses may partially offset these effects [23]. The biological mechanisms underlying this attenuated vaccine response are complex and involve chronic substance-induced inflammation and immune dysregulation, which can compromise the generation of long-lasting immune memory. These alterations raise concerns regarding the duration of vaccine protection in individuals with AUD or TUD. Despite the growing evidence of immune dysfunction, real-world data on COVID-19 vaccine effectiveness in these populations remain limited [22,24].

Although vaccination remains relevant for vulnerable groups, its real-world effectiveness in large population-based cohorts, particularly in the Global South, has not been comprehensively characterized. The analysis of robust Brazilian data offers a unique opportunity to assess the impact of immunization in a diverse and highly exposed population. In this context, the present study aimed to analyze the vaccine effectiveness (VE) of COVID-19 vaccines against mortality in patients diagnosed with AUD and TUD in Brazil.

## 2. Materials and Methods

### 2.1. Study Design, Participants, and Data Sources

**Figure 1:**
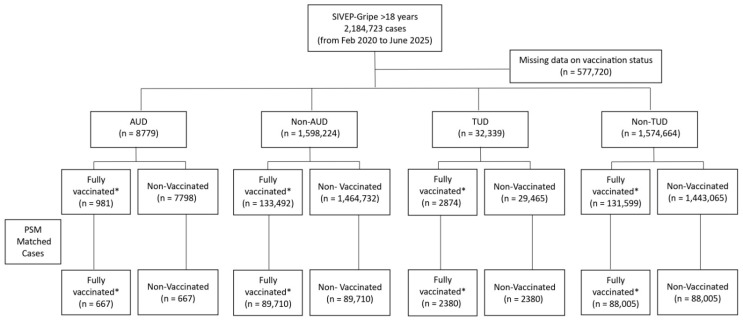
Flowchart of cohort selection. * Individuals who had received three or more doses of a COVID-19 vaccine.

We carried out a retrospective cohort study using Brazilian data from the official national COVID-19 surveillance systems managed by the Ministry of Health, specifically the SIVEP-Gripe database, which monitors patients hospitalized with influenza-like symptoms. The study included all individuals aged 18 years and older with confirmed SARS-CoV-2 infection recorded in this system from 20 February 2020 to 30 June 2025. Confirmation of SARS-CoV-2 infection was based on a positive result from either a quantitative reverse transcription PCR (RT-qPCR) or an antigen test. Additional details about these databases can be found at https://opendatasus.saude.gov.br/dataset (accessed on 30 June 2025). The data management and processing procedures have been previously detailed [25]. The case selection process is depicted in the flowchart shown in Figure 1.

### 2.2. Exposure of Interest

This study evaluated two primary exposures: AUD and TUD. These conditions were identified using dedicated comorbidity fields in the SIVEP-Gripe database based on patient self-report at hospital admission. This information was completed in an accompanying text-filed by healthcare professionals that included clinical evidence of the substances abuse in more detail.

### 2.3. Covariates

We gathered demographic information, which included age (treated as a continuous variable), sex, and geographic location. The geographic areas were categorized based on Brazil’s five official macro-regions, each defined by unique historical and cultural developments, socioeconomic characteristics, and healthcare system frameworks. The clinical information collected encompassed the date when COVID-19 symptoms first appeared, the date of hospital admission, initial signs and symptoms, and the presence of chronic health conditions. Comorbidities were identified through specific fields in the original dataset, indicating whether chronic medical conditions such as asthma, obesity, immunodeficiency, cancer, and diseases of the heart, lungs, kidneys, nervous system, and blood were present or absent. For analysis, the burden of comorbidities was classified according to the number of conditions (none, one, two, or three or more). Due to the nature of the national surveillance databases, missing data in the comorbidity fields were assumed to indicate the absence of a condition. We also considered the period of hospital admission as a covariate, divided into two pandemic phases: the pandemic period (1 February 2020 to 31 December 2022) and the post-pandemic period (1 January 2023 to 30 June 2025).

### 2.4. Vaccination Status (Intervention)

Vaccination status was determined based on the guidelines from the Brazilian Ministry of Health. Individuals who had received three or more doses of a COVID-19 vaccine were deemed fully vaccinated, aligning with the complete vaccination schedule defined during the study period. Although vaccination guidelines changed over time, we defined full vaccination as three doses to align with the complete immunization schedule established from 2022 onwards, when booster doses became widely recommended for adults [18]. Those without any recorded doses were considered unvaccinated. Participants with an incomplete vaccination schedule (one or two doses) were excluded from the analysis as they did not fit the criteria for either comparison group. The threshold of three doses for full vaccination was chosen due to evidence indicating that this regimen offers better and more lasting protection against severe outcomes than two doses, especially in the context of emerging variants and among vulnerable populations.

### 2.5. Outcomes

The primary outcome was mortality, defined as in-hospital death occurring during the same hospitalization episode for confirmed COVID-19. Mortality data were extracted directly from the SIVEP-Gripe database, which records the outcome of each hospitalized case (discharge or death).

### 2.6. Statistical Analysis

Continuous variables were described as medians with interquartile ranges (IQR) and means with standard deviations (SD), whereas categorical variables were represented as proportions. Propensity score matching (PSM) was performed with vaccination status (three or more doses versus unvaccinated) as the exposure variable, divided into four groups based on the presence or absence of alcohol use disorder (AUD) and TUD. The matching process considered age, sex, geographic region, admission period, and number of comorbidities, employing a 1:1 nearest-neighbor method without replacement, with a caliper of 0.2 standard deviations of the propensity score [26,27]. Following matching based on the estimated propensity score, the balance between the vaccinated and unvaccinated groups was assessed using standardized mean differences (SMD) and by visually inspecting propensity score distributions through density plots [28]. From the matched data, the odds ratio for the link between vaccination and outcome was determined using the ratio of discordant pairs. The McNemar test was used to evaluate the statistical significance of the paired-sample analysis. After PSM, vaccine effectiveness (VE) was estimated by comparing the outcome (death) between matched vaccinated and unvaccinated groups, calculated as (1 − odds ratio) × 100%, with 95% CIs derived from the odds ratio CIs. The average treatment effect (ATE) and number needed to vaccinate (NNV) to prevent one death were subsequently derived from the matched samples. All analyses were conducted using R software (version 4.3.0, The R Foundation), and the MatchIt package was used for propensity score matching. STATA (version 19) was used to compute the ATE and NNV. Statistical significance was set at *p* < 0.05.

### 2.7. Ethical Aspects

The need for informed consent was waived because the study used de-identified secondary data sourced from publicly accessible databases. The Ethics Committee of the Federal University of Minas Gerais approved this study (protocol code 6.127.414, 19 June 2023).

## 3. Results

This study analyzed a cohort of 2,184,723 cases from the SIVEP-Gripe database, including 12,115 patients with AUD and 45,679 patients with TUD. The mean age was 60.0 years in the non-exposed group, 58.8 years among individuals with AUD, and 65.4 years among those with TUD, indicating a slightly lower mean age in the AUD group and a higher mean age in the TUD group than in the reference population. Patients with AUD and TUD were predominantly male, accounting for 88.7% and 65.9% of the cases, respectively. Most cases were concentrated in the Southeast, South, and Northeast regions, reflecting the general population distribution across Brazil’s geographic macro-regions. The majority of cases occurred during the pandemic period. Regarding comorbidity burden, the proportion of individuals with three or more comorbidities was higher among patients with AUD (10.2%) and TUD (16.4%) than among their counterparts without these conditions (8.6% and 8.4%, respectively). Regarding the vaccination schedule, the prevalence of individuals who received three or more vaccine doses was higher in the AUD group (11.2%), whereas the TUD and non-exposed groups showed similar prevalence rates (8.9% and 8.4%, respectively). Mortality rates were higher among the exposed groups (46.8% in AUD and 43.1% in TUD) than among their counterparts without these conditions (31.7% for non-AUD and 31.5% for non-TUD) (Table 1).

Table 2 presents the clinical and demographic characteristics of the sample after propensity score matching. As expected, after matching, the possible confounding covariates were evenly distributed across the groups. Notably, the distribution of deaths by vaccination status revealed the protective effect of immunization. Within the AUD group, among those who died, 57.2% were unvaccinated, and 42.8% had received three or more doses. This pattern was even more pronounced in the TUD group: of the patients who died, 60.1% were unvaccinated compared to 39.9% who were fully vaccinated. A similar pattern was observed in non-exposed populations. In the non-AUD group, among those who died, 63.0% were unvaccinated and 37.0% were fully vaccinated. In the non-TUD group, the distribution was nearly identical: 63.2% of deaths occurred among unvaccinated individuals versus 36.8% among fully vaccinated individuals.

We assessed the effectiveness of the complete vaccination regimen using PSM. Post-matching, we analyzed the treatment effect of vaccination on the mortality rate by estimating the VE and calculating the number needed to vaccinate (NNV) across the groups. Among individuals with AUD, the VE was 42% (95% CI: 27.5–53.5), with an NNV of 8 (95% CI: 6–15). In the non-AUD group, the VE was substantially higher (58.5%; 95% CI: 57.6–59.4), with an NNV of 8 (95% CI: 8–9). Regarding TUD, patients presented a VE of 52.6% (95% CI: 46.5–58.1) and an NNV of 8 (95% CI: 7–12). In contrast, individuals without TUD showed a VE of 58.9% (95% CI: 58.0–59.8) and an NNV of 8 (95% CI: 8–9). All estimates were statistically significant (*p* < 0.001).

These findings indicate that, while vaccination was associated with significant protection across all subgroups, the VE was notably lower among individuals with AUD and TUD than among their counterparts without these conditions, despite similar NNV estimates. The ATE values remained consistent across groups, suggesting a comparable absolute risk reduction attributable to vaccination (Table 3).

## 4. Discussion

### 4.1. Key Points

In this large retrospective cohort study based on Brazilian surveillance data, we assessed the protective effect of complete COVID-19 vaccination (three or more doses) against mortality among hospitalized adults with and without alcohol or tobacco use disorders. The analysis included over 2.1 million individuals, of whom approximately 58,000 had a documented diagnosis of AUD or TUD. To ensure comparability between vaccinated and unvaccinated groups, we applied propensity score matching to adjust for demographic characteristics, geographic region, admission period, and comorbidity count. Our results show that although vaccination conferred significant protection across all subgroups, its relative effectiveness was attenuated among individuals with AUD (42%) and TUD (52.6%) compared to those without these conditions (approximately 59%). Nevertheless, the number needed to vaccinate to prevent one death was consistently low (NNV = 8) in all groups, indicating a meaningful absolute benefit even in populations with reduced relative protection. These findings highlight the importance of prioritizing vaccination efforts for individuals with substance use disorders, while also calling attention to the need for additional strategies to enhance vaccine response in this clinically vulnerable population.

### 4.2. Demographic Factors

The male predominance in the AUD (88.7%) and TUD (65.9%) groups reflects the well-known epidemiological patterns of substance use in Brazil and internationally [8,9]. The higher mean age observed in the TUD group (65.4 years) may have contributed to lower VE, as immunological aging (immunosenescence) synergistically interacts with tobacco-induced tissue damage [22]. Although the geographic distribution of cases followed the population concentration in the Southeast and South regions, the persistence of risk patterns after matching reinforces the robustness of the clinical findings, independent of regional disparities in healthcare access.

### 4.3. Clinical Vulnerability and Mortality

The high mortality rate observed in the AUD and TUD groups aligns with international evidence indicating that substance use is an independent predictor of COVID-19 severity. A Danish cohort study documented increased risks of hospitalization [incidence rate ratio (IRR) = 1.72, 95% CI = 1.51–1.95] and 60-day mortality [mortality rate ratio (MRR) = 2.35, 95% CI = 1.94–2.85] among individuals with AUD [8]. In line with these findings, a US-based study reported that individuals with SUD were 13% to 29% more likely to experience COVID-19 reinfection and had significantly higher 30-day mortality [29].

A meta-analysis [30], published early in the pandemic, including 32,849 hospitalized COVID-19 patients, found that current smokers had a significantly increased risk of severe or critical disease [Risk Ratio (RR): 1.98; 95% CI: 1.16–3.38]. Patients with any smoking history also showed higher risks of severe COVID-19 (RR: 1.31; 95% CI: 1.12–1.54), in-hospital mortality (RR: 1.26; 95% CI: 1.20–1.32), disease progression (RR: 2.18; 95% CI: 1.06–4.49), and need for mechanical ventilation (RR: 1.20; 95% CI: 1.01–1.42).

Interestingly, a study [20] reported an apparent protective effect of current smoking against severe COVID-19 outcomes, particularly among lighter smokers. However, this finding should be interpreted with caution because of the potential residual confounding and complex relationship between smoking and inflammatory pathways. The study also suggested that former smokers had a greater risk of severe outcomes, indicating the possible long-term effects of prolonged tobacco use. Our data extend these findings to the Brazilian context, demonstrating that this vulnerability persists in settings characterized by high disease burden and regional inequalities. The higher proportion of individuals with three or more comorbidities in the exposed groups suggests that accumulated biological frailty may potentiate the severity of viral infection [22].

In addition to their known biological effects on the immune response, AUD and TUD may increase vaccine hesitancy. Havelka et al. [31], in a systematic review of 103 studies, found that tobacco use was associated with poorer vaccine acceptance, uptake, and adherence across multiple vaccines, while alcohol and drug use were linked to similarly suboptimal outcomes. Additionally, Powell et al. [32] showed that substance use-related stigma toward opioids, methamphetamine, and cocaine was positively associated with COVID-19 vaccine hesitancy, although no significant association was found for alcohol use-related stigma. In contrast, in our study, the vaccination rates were similar between the TUD and non-TUD groups and were slightly higher in the AUD group than in the non-AUD group.

### 4.4. Reduced Vaccine Effectiveness and Immunological Mechanisms

The reduced VE observed in this study among patients with AUD (42%) and TUD (52.6%) is consistent with research indicating an attenuated immune response in these populations. For context, a Cochrane systematic review by Graña et al. [33] reported that WHO-approved vaccines (e.g., BNT162b2, mRNA-1273, ChAdOx1) reduced confirmed symptomatic COVID-19 by 67% to 98% and severe or critical disease by 76% to 99% compared to placebo, with high-certainty evidence. Similarly, a meta-analysis [34] of 11 RCTs involving 247,186 participants confirmed significant protection against COVID-19 incidence in vaccinated individuals compared to placebo, with mRNA-based vaccines showing the highest efficacy, followed by inactivated and nonreplicating viral vector vaccines. Our finding of lower VE in AUD and TUD suggests that these populations do not achieve the same level of protection as the general population, despite receiving the same vaccine regimens.

For AUD, the immunological impact appears to be both dose-dependent and closely linked to the presence of alcohol-related liver disease, which is known to impair T and B cell function and reduce antibody production [35]. Chronic alcohol consumption has been associated with lower peak antibody titers following primary vaccination and an accelerated waning of humoral immunity over time, although booster doses may partially mitigate this difference [24]. These findings align with our observed vaccine effectiveness estimates, suggesting that even in the presence of vaccination, the immunological threshold for protection is either lower or less sustained in individuals with substance use disorders. This blunted or more rapidly declining immune response may help explain the higher incidence of severe outcomes observed among vaccinated individuals in these high-risk groups, reinforcing the need for tailored immunization strategies, including closer monitoring of antibody persistence and prioritized booster scheduling [23].

Chronic alcohol consumption disrupts both innate and adaptive immune responses through multiple mechanisms. It compromises the function of alveolar macrophages and dendritic cells, impairs the migration of leukocytes, and induces a state of low-grade systemic inflammation, marked by altered cytokine expression [36,37]. This baseline immune dysregulation is critical because COVID-19 vaccines, particularly mRNA and viral vector platforms, rely on endogenous synthesis of the SARS-CoV-2 spike protein to mount a robust and durable adaptive response. Furthermore, it has been hypothesized that alcohol consumption may exacerbate the so-called spike protein-related effect [38,39]. By inducing the overexpression of the ACE2 receptor and activating pro-inflammatory pathways, alcohol could theoretically amplify the inflammatory response to the vaccine’s spike protein, potentially leading to a less efficient development of long-term immunological memory and contributing to the more rapid waning of antibodies observed in these patients [40,41].

Among smokers, studies have confirmed that anti-SARS-CoV-2 IgG antibody levels are lower after complete vaccination than in non-smokers [22,24]. Chronic smoking induces a state of low-grade systemic inflammation associated with persistent activation of pro-inflammatory pathways and dysfunction of B and T cells. Continuous exposure to toxic components of cigarettes may also impair the formation of memory cells and antibody affinity maturation, resulting in less robust and shorter-lived vaccine responses. Nicotine appears to exert direct immunomodulatory effects by inhibiting lymphocyte proliferation and the production of cytokines that are essential for adaptive immune responses. Furthermore, an accelerated decline (waning) of immunity over time has been observed in this population, which may compromise long-lasting protection against severe disease [22,42]. This synergy between pre-existing immune dysfunction and a potentially heightened inflammatory response to the vaccine antigen offers a compelling explanation for lower vaccine effectiveness and the need for prioritized booster doses in this vulnerable population.

### 4.5. Implications for Health Policies

These findings validate and extend previous international studies, demonstrating high external validity for hospitalized COVID-19 populations with demographic profiles similar to those in Brazil. The consistency of the NNV across groups (NNV = 8) is a crucial public health finding, demonstrating that vaccination is equally efficient in terms of lives saved per vaccinated individual, even among individuals with different baseline health conditions. This justifies prioritizing active outreach and booster vaccination strategies for individuals with AUD and TUD, who often face access barriers and greater stigma in health care services [31,32].

### 4.6. Limitations and Strengths

This study has limitations inherent by using secondary data analysis. Our study relied on a large-scale administrative healthcare dataset, which, while providing substantial sample sizes and broad population coverage, has limitations. First, missing data is a well-recognized issue in administrative databases. Although SIVEP-Gripe is a mandatory national surveillance system that is considered reliable for hospitalization data, approximately 25% of cases lack vaccination status due to the late addition of this field, system update delays, high workload during the peak of the pandemic, and underreporting in resource-limited regions. Second, the identification of AUD and TUD relied on the availability and accuracy of comorbidity fields in the SIVEP-Gripe database, which lacked detailed information on severity, duration of use, or clinical history. This may have led to underreporting of these exposures and precluded the inclusion of critical features, such as patterns of substance use or alcohol-related liver disease severity. Third, the data on comorbidities, including AUD and TUD, were based on self-report rather than clinical confirmation, which may introduce recall and social desirability biases, as individuals may underreport substance use due to stigma. Fourth, despite propensity score matching, residual confounding from unmeasured variables (e.g., nutritional status, detailed socioeconomic indicators, and exact time since the last vaccine dose) cannot be entirely ruled out. Fifth, the study spanned different viral variants and vaccine platforms, which may have heterogeneously influenced the VE estimates. Sixth, the generalizability of the findings may be limited, as the study was conducted in a single middle-income country. Seventh, selection bias may be present, as the SIVEP-Gripe database only includes hospitalized patients, excluding those with mild or asymptomatic COVID-19 who did not seek medical care. Finally, the absence of comparable post-pandemic studies restricts external comparisons.

Nevertheless, some noteworthy strengths must be highlighted, including the large cohort size, which enabled stratified analyses of AUD and TUD subgroups; the use of robust methods to estimate VE, ATE, and NNV; and the use of real-world national data covering both the pandemic and post-pandemic periods. To our knowledge, this is among the first studies to rigorously quantify vaccine effectiveness and NNV specifically in individuals with alcohol and tobacco use disorders in a middle-income country.

## 5. Conclusions

This study showed that individuals with AUD and TUD are at substantially higher risk of fatal COVID-19 outcomes than the general population, with unvaccinated patients within these groups facing an elevated mortality risk. Although vaccine effectiveness against mortality was significantly lower among patients with AUD and TUD than among their non-exposed counterparts, the absolute benefit of vaccination remained substantial, as evidenced by a stable number of needed to vaccinate across all groups and the finding that the majority of deaths in both substances use disorders occurred among unvaccinated individuals. These findings highlight the severe course of COVID-19 in patients with AUD and TUD, confirming that they represent a high-risk population. Clinical interventions should focus not only on primary immunization but also on maintaining high antibody titers through timely booster doses and integrated management of comorbidities in these vulnerable patients.

## Figures and Tables

**Table 1:** Clinical and demographic features of adults with confirmed COVID-19 categorized by the presence of alcohol use disorder (AUD) and tobacco use disorder (TUD) before PSM (n = 2,184,723).

Covariates *	AUD Cohort (%) 12,115 (0.6)	Non-AUD Cohort (%) 2,172,475 (99.4)	TUD Cohort (%) 45,679 (2.1)	Non-TUD Cohort (%) 2,138,896 (97.9)
Age (years)				
Median (IQR)	59.0 (49.4–58.6)	60.5 (46.9–73.6)	66.3 (57.2–75.2)	60.3 (46.7–73.5)
Mean (SD)	58.8 (13.8)	60.2 (17.6)	65.4 (13.9)	60.1 (17.6)
Sex (n = 2,184,590)				
Male	10,751 (88.7)	1,185,782 (54.6)	30,120 (65.9)	1,166,413 (54.5)
Female	1364 (11.3)	986,693 (45.4)	15,574 (34.1)	972,483 (45.5)
Region				
Southeast	4556 (37.6)	1,079,740 (49.7)	16,076 (35.2)	1,068,220 (49.9)
South	2920 (24.1)	372,879 (17.2)	12,578 (27.5)	363,221 (17.0)
Central-West	1178 (9.7)	221,022 (10.2)	4094 (9.0)	218,106 (10.2)
Northeast	3213 (26.5)	357,993 (16.5)	11,942 (26.1)	349,264 (16.3)
North	248 (2.0)	140,974 (6.5)	1007 (2.2)	140,215 (6.6)
Period (n = 1,715,308)				
Pandemic	11,267 (93)	2,099,069 (96.6)	44,877 (98.2)	2,065,459 (96.6)
Post pandemic	848 (7.0)	73,539 (3.4)	820 (1.8)	73,567 (3.4)
Number of Comorbidities				
None	4640 (38.3)	920,665 (42.4)	10,985 (24)	914,310 (42.7)
One	3732 (30.8)	637,653 (29.3)	14,747 (32.3)	626,638 (29.3)
Two	2504 (20.7)	428,504 (19.7)	12,460 (27.3)	418,548 (19.6)
Three or more	1239 (10.2)	185,796 (8.6)	7505 (16.4)	179,530 (8.4)
Vaccine schedule (n = 1,607,003)				
None	7798 (88.8)	1,464,732 (91.6)	29,465 (91.1)	1,443,065 (91.6)
Three or more	981 (11.2)	133,492 (8.4)	2874 (8.9)	131,599 (8.4)
Death (total)				
No	6446 (53.2)	1,443,065 (91.6)	25,987 (56.9)	1,464,243 (68.5)
Yes	5669 (46.8)	688,824 (31.7)	19,710 (43.1)	674,783 (31.5)

* Data (n) in the first column represent the available data for those covariates with missing values (gender, period, and vaccine).

**Table 2:** Clinical and demographic features of adults with confirmed COVID-19 categorized by the presence of alcohol use disorder (AUD) and tobacco use disorder (TUD) after PSM.

Covariates	AUD (%) 1334 (100)		Non-AUD (%) 179,420 (100)		TUD (%) 4760 (100)		**Non-TUD (%)** **176,010 (100)**	
	Unvaccinated 667 (50.0)	Fully Vaccinated 667 (50.0)	Unvaccinated 89,710 (50.0)	Fully Vaccinated 89,710 (50.0)	Unvaccinated 2380 (50.0)	Fully Vaccinated 2380 (50.0)	**Unvaccinated** **88,005 (50.0)**	**Fully Vaccinated** **88,005 (50.0)**
Age (years)								
Median (IQR)	64.0 (54–74)	65.0 (56–73)	75.0 (64–85)	76.0 (64–85)	73.0 (64–81)	72.0 (65–81)	75.0 (64–85)	76.0 (64–85)
Mean (SD)	64.1 (13.2)	64.2 (13.2)	72.4 (16.7)	72.6 (16.9)	72.2 (12.5)	71.9 (12.2)	72.5 (16.8)	72.5 (16.9)
Sex								
Male	580 (49.7)	586 (50.3)	41,711 (49.3)	46,816 (49.4)	1414 (49.1)	1463 (50.9)	40,370 (49)	42,049 (51.0)
Female	87 (51.8)	81 (48.2)	47,999 (50.6)	42,894 (50.7)	966 (51.3)	917 (48.7)	47,635 (50.9)	45,956 (49.1)
Region								
Southeast	275 (50.6)	269 (49.4)	52,289 (50.3)	51,754 (49.7)	850 (48.0)	922 (52.0)	51,124 (50.1)	50,986 (49.9)
South	200 (49.1)	207 (50.9)	19,247 (52.1)	17,679 (47.9)	907 (50.4)	891 (49.6)	17,930 (51.2)	17,118 (48.8)
Central-West	71 (54.2)	60 (45.8)	6714 (48.5)	7118 (51.5)	163 (52.8)	146 (47.2)	7066 (50.2)	7006 (49.8)
Northeast	110 (47.2)	123 (52.8)	9414 (46.1)	11,023 (53.9)	447 (52.3)	407 (47.7)	9674 (47.3)	10,759 (52.7)
North	11 (57.9)	8 (42.1)	2046 (48.9)	2136 (51.1)	13 (48.1)	14 (51.9)	2211 (50.9)	2136 (49.1)
Period								
Pandemic	559 (50.0)	559 (50.0)	83,474 (50.0)	83,360 (50.0)	2322 (50.0)	2321 (50.0)	81,706 (50.0)	81,591 (50.0)
Post pandemic	108 (50.0)	108 (50.0)	6236 (49.5)	6350 (50.5)	58 (49.6)	59 (50.4)	6299 (49.5)	6414 (50.5)
Number of Comorbidities								
None	186 (50.4)	183 (49.6)	25,973 (51.0)	24,954 (49.0)	363 (52.4)	330 (47.6)	25,904 (50.9)	24,992 (49.1)
One	202 (49.3)	208 (50.7)	28,334 (49.6)	28,829 (50.4)	712 (49.2)	735 (50.8)	28,314 (50.1)	28,221 (49.9)
Two	169 (48.3)	181 (51.7)	23,298 (50.1)	23,185 (49.9)	819 (50.8)	792 (49.2)	22,183 (49.6)	22,574 (50.4)
Three or more	110 (53.7)	95 (46.3)	12,105 (48.7)	12,742 (51.3)	486 (48.2)	523 (51.8)	11,604 (48.7)	12,218 (51.3)
Death								
No	313 (43.8)	402 (56.2)	48,471 (42.5)	65,538 (57.5)	1123 (42.1)	1544 (57.9)	47,458 (42.4)	64,415 (57.6)
Yes	354 (57.2)	265 (42.8)	41,239 (63.0)	24,172 (37.0)	1257 (60.1)	836 (39.9)	40,547 (63.2)	23,590 (36.8)

**Table 3:** McNemar test results for paired samples after Propensity Score Matching: Vaccine Effectiveness (VE), Average Treatment Effect (ATE), and Number Needed to Vaccinate (NNT), stratified by Alcohol Use Disorder (AUD) and Tobacco Use Disorder (TUD).

AUD	Non-Vaccinated (Survival)	Non-Vaccinated (Death)	PSM VE (%) (95% CI)	ATE (95% CI)	NNV (95% CI)	*p*-Valor
Vaccinated (survival)	190	212	42 (27.5–53.5)	−0.118493 (−0.064876; −0.172109)	8 (6–15)	<0.001
Vaccinated (death)	123	142				
Non-AUD						
Vaccinated (survival)	36,358	29,180	58.5 (57.6–59.4)	−0.119362 (−0.114134; −0.124591)	8 (8–9)	<0.001
Vaccinated (death)	12,113	12,059				
TUD						
Vaccinated (survival)	744	800	52.6 (46.5–58.1)	−0.118993 (−0.085968; −0.152019)	8 (7–12)	<0.001
Vaccinated (death)	379	457				
Non-TUD						
Vaccinated (survival)	35,618	28,797	58.9 (58–59.8)	−0.119617 (−0.114539; −0.124694)	8 (8–9)	<0.001
Vaccinated (death)	11,840	11,750				

## Data Availability

The original data presented in the study are openly available in https://opendatasus.saude.gov.br/dataset/ (accessed on 30 June 2025). Our analysis code is available upon request from the corresponding author (Eduardo A. Oliveira, eduolive812@gmail.com).

## Abbreviations

The following abbreviations are used in this manuscript:

COVID-19	Coronavirus disease 2019
SARS-CoV-2	Severe acute respiratory syndrome coronavirus 2
AUD	Alcohol Use Disorder
TUD	Tobacco Use Disorder
VE	Vaccine Effectiveness
SIVEP-Gripe	Influenza Epidemiological Surveillance Information System
RT-qPCR	Reverse Transcription quantitative Polymerase Chain Reaction
IQR	Interquartile Range
SD	Standard Deviation
PSM	Propensity score matching
SMD	Standardized mean differences
CI	Confidence Interval
ATE	Average Treatment Effect
NNV	Number Needed to Vaccinate
IRR	Incidence Rate Ratio
MRR	Mortality Rate Ratio
RR	Risk Ratio
mRNA	messenger Ribonucleic Acid
ACE2	Angiotensin-Converting Enzyme 2
IgG	Immunoglobulin G
